# Supplementary material for: The Construction and Exploration of a Comprehensive MicroRNA Centered Regulatory Network in Foxtail Millet (Setaria italica L.)
Source: Front Plant Sci. 2022 May 6;13:848474. doi: 10.3389/fpls.2022.848474 (PMC9121102; doi:10.3389/fpls.2022.848474)

T=Seita.5G343700.1_Q=Sit−miRN3027_S=1080

category=2_p=0.60836518784234

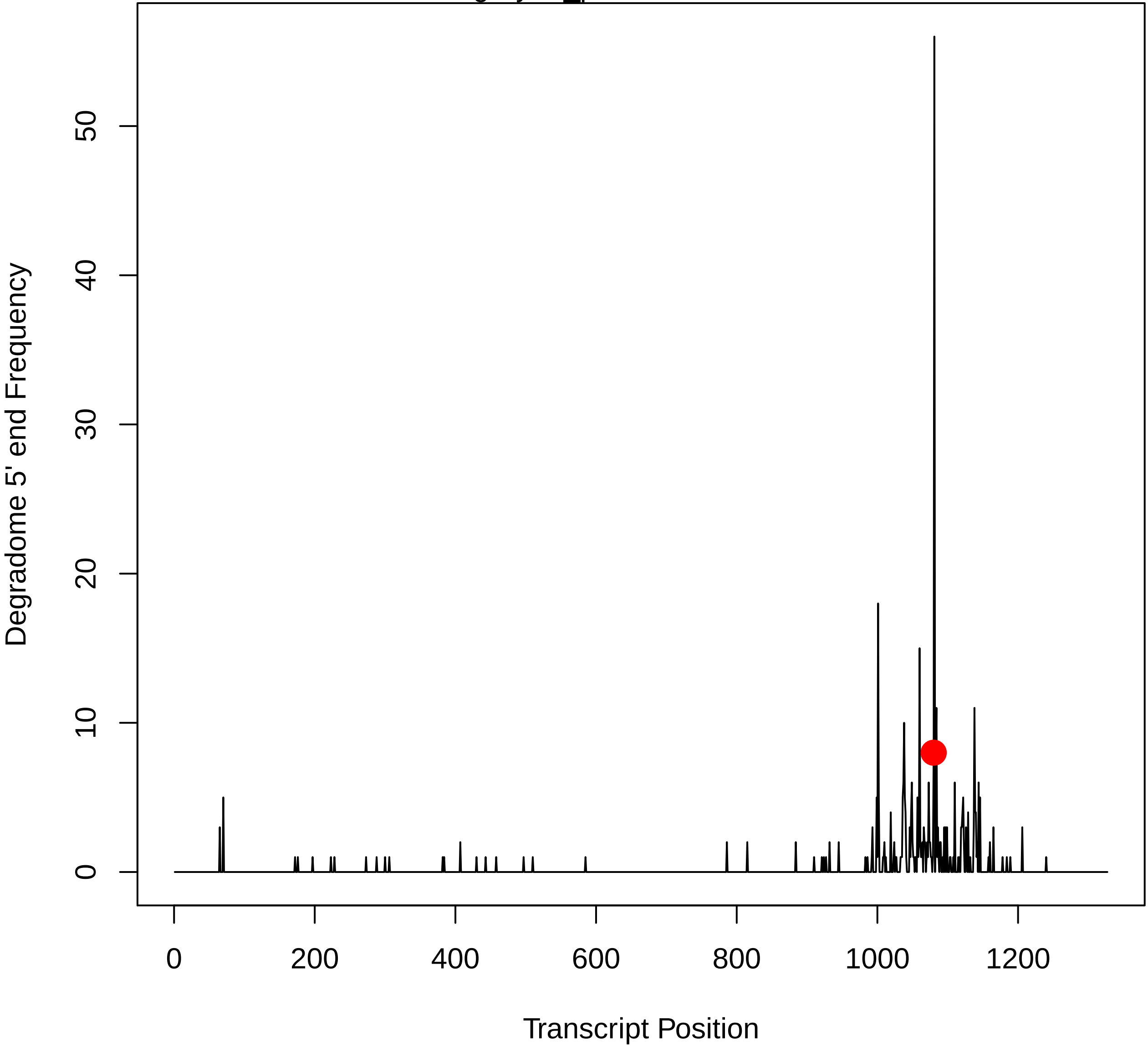

Supplement: Supplementary file 3 [file Data_Sheet_3.zip › Sit-miRN3027_Seita.5G343700.1_1080_TPlot.pdf]
